# PET Tracers for Clinical Imaging of Breast Cancer

**DOI:** 10.1155/2012/710561

**Published:** 2012-08-29

**Authors:** Iván Peñuelas, Inés Domínguez-Prado, María J. García-Velloso, Josep M. Martí-Climent, Macarena Rodríguez-Fraile, Carlos Caicedo, María Sánchez-Martínez, José A. Richter

**Affiliations:** Department of Nuclear Medicine, University Clinic of Navarra, Avenida Pío XII 36, 31008 Pamplona, Spain

## Abstract

Molecular imaging of breast cancer has undoubtedly permitted a substantial development of the overall diagnostic accuracy of this malignancy in the last years. Accurate tumour staging, design of individually suited therapies, response evaluation, early detection of recurrence and distant lesions have also evolved in parallel with the development of novel molecular imaging approaches. In this context, positron emission tomography (PET) can be probably seen as the most interesting molecular imaging technology with straightforward clinical application for such purposes. Dozens of radiotracers for PET imaging of breast cancer have been tested in laboratory animals. However, in this review we shall focus mainly in the smaller group of PET radiopharmaceuticals that have lead through into the clinical setting. PET imaging can be used to target general metabolic phenomena related to tumoural transformation, including glucose metabolism and cell proliferation, but can also be directed to specific hormone receptors that are characteristic of the breast cancer cell. Many other receptors and transport molecules present in the tumour cells could also be of interest for imaging. Furthermore, molecules related with the tumour microenvironment, tumour induced angiogenesis or even hypoxia could also be used as molecular biomarkers for breast cancer imaging.

## 1. Introduction

PET is a molecular imaging modality in which compounds labelled with positron emitting radioisotopes are used to measure biological processes with only trace amounts of the radiolabelled probes. Molecules labelled with positron-emitting radionuclides are retained in tissues as a result of binding to a receptor, or cell entrapment owing to enzyme-catalyzed conversion after uptake by a cell membrane transporter. Tomographic images of the distribution of the radioactivity within the body can be generated and quantitatively evaluated by coincidence detection of the gamma rays resulting from the mutual annihilation of a positron emitted by the radionuclide and an electron of a nearby atom.

PET radiotracers are molecular probes that can be designed and synthesized to target very many different molecular and cellular events. In the specific case of breast cancer, there is a wide panoply of specific and general targets one could think about. However, being there a great number of molecules labelled with different positron emitting radionuclides (see Table 1) that have been synthesised and tested in vivo in laboratory animals, only a few of them have lead through into the clinical setting. In this review, we shall focus mainly in this smaller group of radiotracers. Papers focused in PET biomarkers for pre-clinical studies can be found elsewhere.

## 2. Glucose Metabolism

Although the number of PET tracers synthesised and tested *in vivo* largely exceeds 300, only a fraction has been used in humans. In addition, probably more than 90% of the clinical PET studies are performed with the analogue of glucose 2-[^18^F]-fluoro-2-deoxy-D-glucose (FDG). Uptake and metabolism of FDG within tumour cells is higher than in normal tissue because of increased glycolysis. The incorporation of FDG to the neoplastic cells is favoured by the increase of glucose membrane transporters (GLUTs) which are overexpressed in breast cancer due to the activation of their genes. FDG is phosphorylated to FDG-6-phosphate by hexokinase-1, which also has increased activity in tumour cells due both to genetic and allosteric modifications. Therefore, the detected concentration of labelled FDG is proportional to the uptake and metabolism of glucose.

In patients with breast cancer, FDG PET/CT results in improved sensitivity for the detection of both lymph node infiltration and distant metastases compared to conventional imaging techniques [1]. Therefore, FDG PET/CT offers clinically relevant information on the staging of patients with breast cancer, has prognostic value, and enables the assessment of therapeutic response and relapse. The development of PET scanners dedicated to breast (positron emission mammography) has improved spatial resolution and sensitivity, allowing its clinical application in the study of the primary tumour [2].

Tumour uptake of FDG is variable and depends not only on tumour size, but also on the histological type and histological grade. The hormone receptor status and other immunohistochemical factors with prognostic value are also relevant, such as p53 and Ki-67 expression. FDG uptake is higher in ductal carcinoma than in lobular carcinoma and in patients with poor prognostic features as high grade and hormone receptor negativity [3].

**Figure 1:**
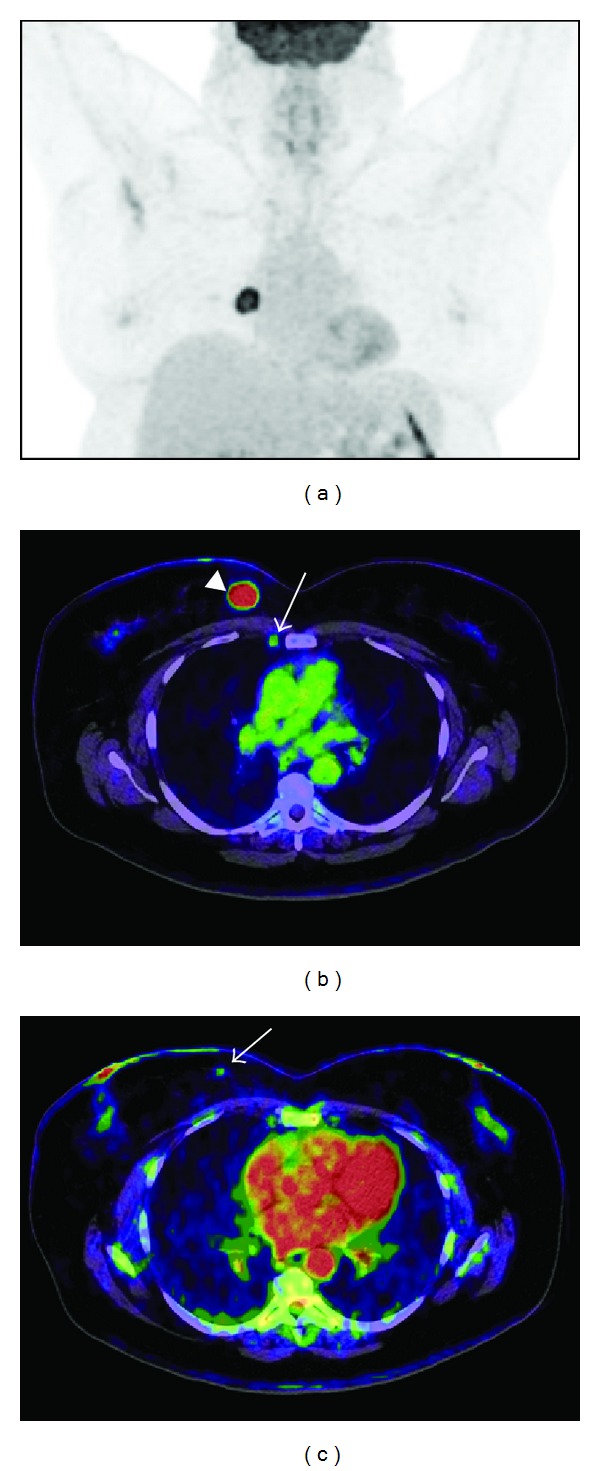
A 60-year-old female (BMI = 37) with a right-sided infiltrating ductal carcinoma was referred for staging. Images were obtained in a Biograph mCT (Siemens) 60 min after i.v. injection of 478 MBq of FDG. (a) FDG-PET maximum intensity projection of the thorax. (b) Axial fused FDG-PET/CT images of the primary tumour in the upper-inner quadrant (arrowhead) (SUV = 5,3), a metastasic lymph node in the right internal mammary chain (arrow) (SUVmax = 1,38) was found in the PET and histopathologically confirmed. (c) Axial fused FDG-PET/CT image of a second tumour in the same breast identified in FDG-PET (arrow) (SUVmax = 0,5), not detected by conventional imaging techniques and biopsy-proven.

Regarding nodal staging, when FDG PET/CT shows axillary uptake, the positive predictive value of metastatic infiltration is greater than 95%. Despite these findings, lymph node uptake should always be confirmed due to the low specificity of FDG. In addition, FDG PET/CT can show disease not detected by conventional imaging techniques in internal mammary chain and mediastinum [4] (Figure 1). However, when there is no FDG uptake in the axilla, given the low sensitivity due to limitations in detecting lymph node micrometastasis and very small tumours, axillary sentinel lymph node biopsy should be performed for a correct staging [5].

**Figure 2:**
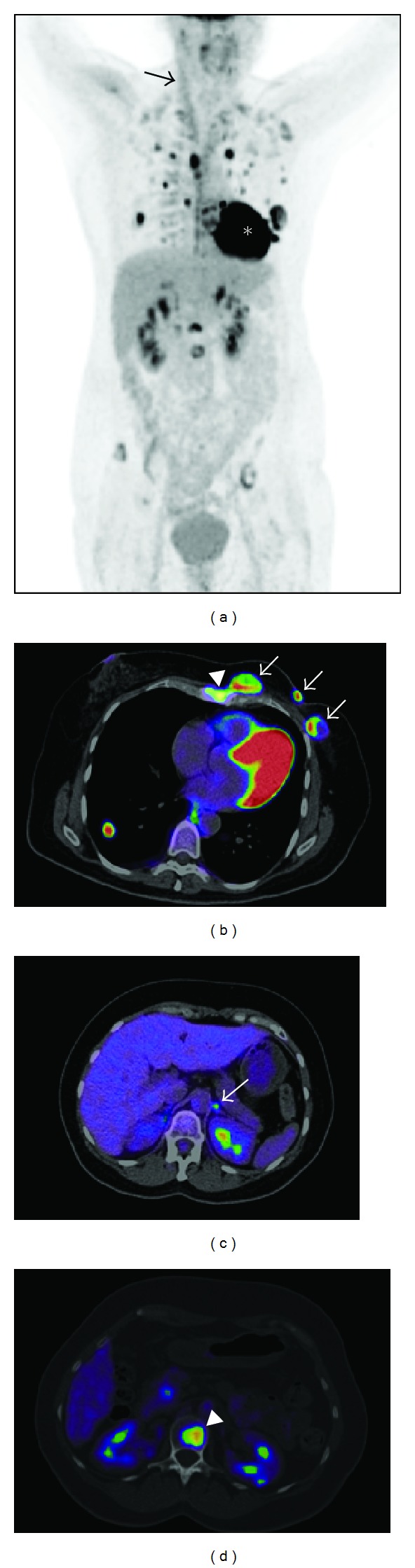
A 47-year-old woman with a left-sided multifocal infiltrating ductal carcinoma, Luminal B, referred for staging. Images were obtained in a Biograph mCT (Siemens) 60 min after i.v. injection of 363 MBq of FDG. (a) FDG-PET whole-body maximum intensity projection: multifocal left-sided breast tumour, lymph node metastasis in the left axilla, left internal mammary chain and mediastinum, left adrenal gland metastasis, multiple bilateral lung metastases, sternal, spine, and pelvic bone metastases. Physiological uptake was identified in paraspinal and supraclavicular fossa brown fat, right sternocleidomastoid muscle (arrow), myocardium (∗), kidneys and bladder. (b) Axial fused FDG-PET/CT image of the multifocal tumour in the left breast (arrows), sternum (arrowhead), and right lung metastasis. (c) Axial fused FDG-PET/CT image of the left adrenal gland metastasis (arrow). (d) Axial fused FDG-PET/CT image of a vertebral metastasis (arrowhead).

FDG PET/CT contributes significantly both in defining the extent of disease and the choice of appropriate therapy in patients with advanced tumours (Figure 2). FDG PET/CT allows early assessment of therapeutic response in patients receiving neoadjuvant therapy, for evaluation of response to new biotherapies and for prediction of outcome [6].

FDG PET/CT is superior to CT in the diagnosis of tumour recurrence in patients with elevated tumour markers, both in sensitivity, specificity, and overall accuracy, with changes in clinical management in 50% of patients [7].

## 3. Proliferation

**Figure 3:**
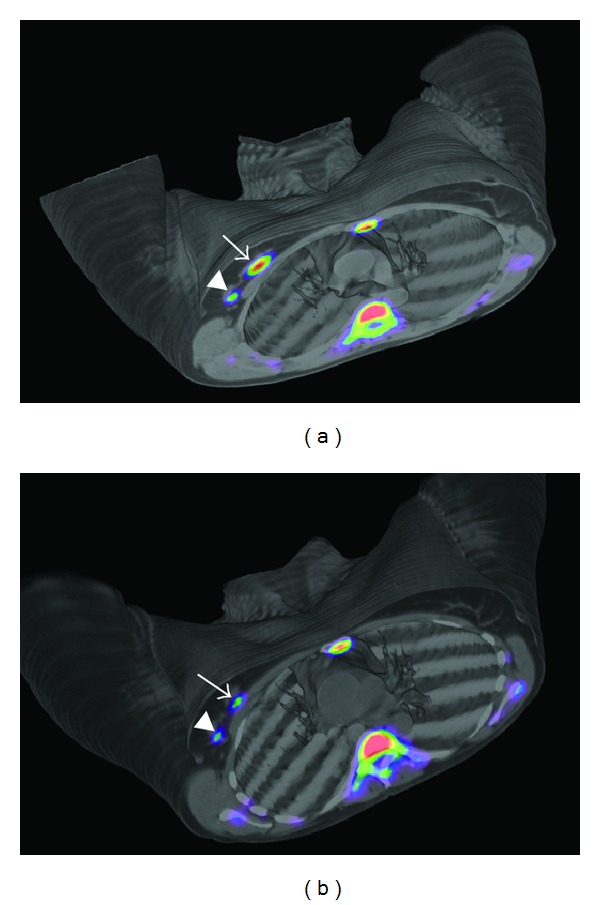
(a) Baseline PET/CT images obtained in a Biograph Duo LSO (Siemens) 75 min after injection of 405 MBq of ^18^FLT in a 47-year-old woman with a right-sided infiltrating ductal carcinoma (SUVmax = 5,42) (arrow) and lymph node uptake (SUVmax = 1,85) (arrowhead). Physiological bone marrow uptake was identified. (b) PET/CT images obtained 75 min after injection of 529 MBq of ^18^FLT after one cycle of neoadjuvant therapy. SUVmax decreased to 3,57 in the primary tumour and to 0,80 in the lymph node, consistent with metabolic response.

One of the main hallmarks of tumour cells is their ability to sustain chronic proliferation, which represents a key target in the scene of the new anticancer therapeutic agents [8]. The study of the proliferation rate is important not only in the initial staging and characterization of the tumour, but also in the assessment of response, both at an early stage (Figure 3) during the initial treatment schedule—as a way to predict the clinical outcome—or at the end of the treatment.

Back to 1998, Shields et al. [9] developed and tested 3′-deoxy-3′-[^18^F]fluorothymidine (^18^FLT) as a biomarker for *in vivo* imaging of cell proliferation, demonstrating that it was resistant to degradation *in vivo*, retained in proliferating tissues by the action of thymidine kinase 1 and produced high-contrast images of normal marrow and tumours.

Although ^18^FLT is not a routine tool in the clinical practice in breast cancer, it may play an important role in the staging, monitoring, and prediction of response to therapy agents [10]. Moreover, ^18^FLT strongly correlates with the immunohistochemical proliferation index ki-67 [11]. ^18^FLT-PET can be of value as an early response predictor for different chemotherapeutic agents. In a prospective study in 20 patients with stages II–IV breast cancer under docetaxel treatment, Contractor et al. [12] aimed to establish biomarkers indicating clinical response to taxanes. Patients underwent a baseline dynamic ^18^FLT-PET scan followed by a scan 2 weeks after initiating the first or second cycle of docetaxel. ^18^FLT-derived PET variables were compared with anatomic response after 3 cycles and concluded that changes in tumour proliferation assessed by ^18^FLT early after initiating docetaxel chemotherapy could predict lesion response with good sensitivity. Furthermore, Kenny et al. [13] demonstrated that ^18^FLT can detect changes in breast cancer proliferation as early as at 1 week after 5-fluorouracil, epirubicin, and cyclophosphamide therapy. Decreases in the irreversible trapping constant and the standardized uptake value (SUV) at 1 week discriminated between clinical response and stable disease. In an effort to find simplified valuable ^18^FLT-PET uptake measures, Lubberink et al. [14] studied with a dynamic ^18^FLT-PET scan 15 patients with locally advanced breast cancer both prior to and after the first cycle of chemotherapy with fluorouracil, epirubicin or doxorubicin, and cyclophosphamide. The authors concluded that tumour-to-whole blood ratio may be preferred to SUV as a simplified measure for monitoring response.

## 4. Hypoxia

Another important hallmark of cancer disease is hypoxia. This microenvironmental factor facilitates the metastasic spread and is related to poor response to radiation, chemotherapy, genetic instability, and selection for resistance to apoptosis due to its effect on various metabolic, molecular-genetic, and pathophysiologic adaptive processes.

Tumour hypoxia is a condition of insufficient O_2_ to support metabolism and occurs when tumour outgrows its vascularity supply. It has been identified as one of major independent prognostic factors influencing response to therapy and overall survival in many malignancies, including breast cancer [15, 16].

The occurrence of hypoxia in human tumours has in most cases been inferred from histologic findings and from evidence of hypoxia in animal tumour studies. *In vivo* demonstration of hypoxia required tissue measurements with oxygen electrodes and the invasiveness of this technique has limited its applications. Consequently, there has been a growing impetus to develop noninvasive imaging methods to detect and assess tumour hypoxia.

**Figure 4:**
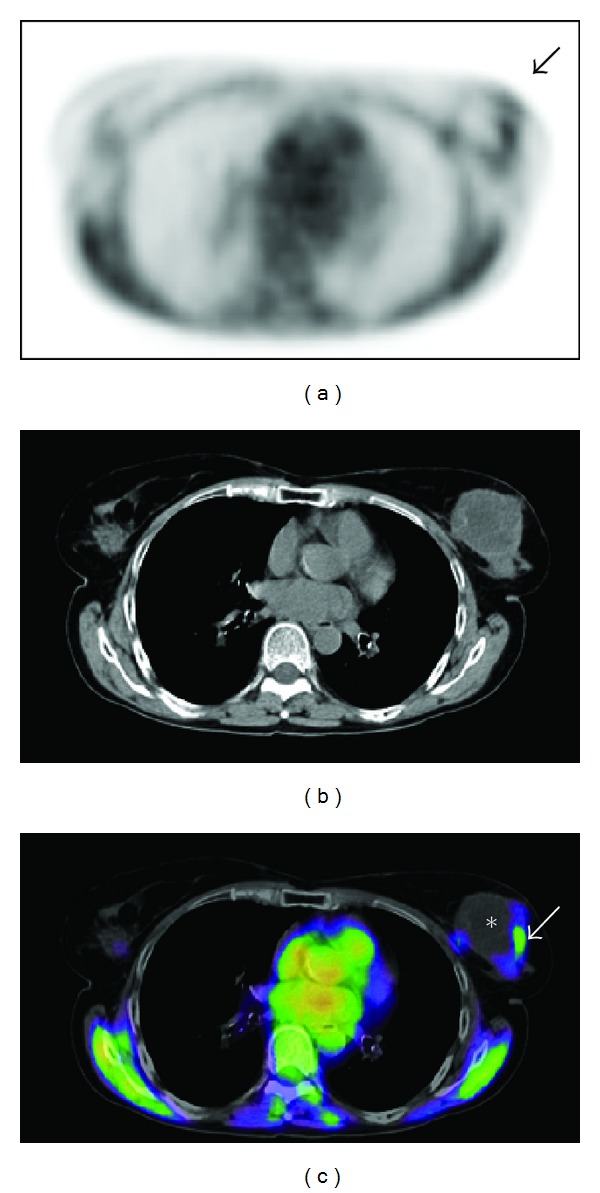
Axial PET (a), CT (b), and fused PET/CT (c) images obtained in a Biograph Duo LSO (Siemens) two hours after i.v. injection of 368 MBq of FMISO, in a 64-year-old woman with a left-sided triple negative infiltrating ductal carcinoma, T2N1M0. Peripheral tumour uptake of FMISO (SUVmax = 1,44) consistent with hypoxia (arrow), and inside tumour lack of activity due to central necrosis (∗) were observed. Physiological uptake in muscles and mediastinum was identified.

The first clinical studies to image hypoxia using PET were based on halogenated tracers of 2-nitroimidazoles, such as [^18^F]fluoromisonidazole (^18^FMISO) [17]. This compound diffuses through cell membranes and when tissular pO_2_ is below 10 mm Hg becomes reduced in viable cells by nitroreductase and once reduced is accumulated intracellularly. Two to four hours after injection, the retention is considered to be specific to cellular hypoxia (Figure 4).

Antiangiogenic therapy has been thought to hold significant potential for the treatment of cancer. However, in the specific case of breast cancer, new research using preclinical models suggests that antiangiogenic agents actually increase invasive and metastatic properties of breast cancer cells by augmenting the population of cancer stem cells by generating intratumoural hypoxia [18]. Albeit no results from human studies have been reported, PET imaging of hypoxia could help shed some light on this cutting-edge story.

Although there are not many published papers on ^18^FMISO in breast cancer, its utility has been well established in other tumours, such as glioblastoma multiforme, rectal, lung and head and neck carcinomas [19].

Rajendran et al. [20] compared the glucose metabolism (FDG-PET) and hypoxia (^18^FMISO-PET) in four different types of tumours, including seven patients with breast cancer. They concluded that although hypoxia is a general factor affecting glucose metabolism, some tumours can have modest glucose metabolism, whereas some highly metabolic tumours are not hypoxic, showing discordance in tracer uptake that can be tumour-type-specific.

## 5. Radiolabelled Choline Derivatives

Transformation from choline to phosphocholine increases with malignant transformation and progression of mammary epithelial cells *in vitro*. Furthermore, increase in phosphocholine can be attributed to expression of the enzyme choline kinase-*α*. It is also important to consider that both choline kinase activity and cellular phosphocholine levels are regulated by the growth factor receptor-MAPK pathway, the same one that modulates estrogen-independent growth [21].

Once the fundamentals for the use of N-[^11^C]methyl-choline (^11^C-choline) were established, Contractor et al. [22] showed that breast tumours were well visualized in 30 of 32 patients with good tumour background ratios, albeit surprisingly a poor association was found with tumour size, estrogen receptor, progesterone receptor, human epidermal growth factor receptor-2, Ki-67, and nodal status.

Albeit the exact biological mechanism of increased ^11^C-choline uptake in certain tumours is unknown, choline is required for membrane synthesis in actively proliferating cells. Consequently, it could be hypothesized that ^11^C-choline uptake should be higher in cells undergoing proliferation. More recently, the same group [23] has demonstrated in a set of 21 patients with estrogen receptor positive breast cancer that choline metabolism assessed by ^11^C-choline PET and proliferation determined by ^18^FLT-PET were correlated in ER-positive breast cancer, concluding that high ^11^C-choline uptake is a measure of cellular proliferation in this setting.

Regarding the use of fluorine labelled choline derivatives in diagnosis of breast cancer, no reports can be found in the literature, apart from an incidental finding in a male patient with elevated PSA levels that was studied by ^18^F-fluorocholine PET for possible diagnosis of prostate cancer [24]. PET/CT images revealed focal uptake in the left breast that was found by biopsy to be an invasive ductal breast carcinoma. Prostate biopsy also revealed prostate cancer corresponding to an area of increased prostatic ^18^F-fluorocholine uptake.

## 6. Estrogen Receptors

Endocrine therapy targeting steroid receptors remains the most effective form of systemic therapy in breast cancer. Thus, receptor ligands such as fluorine-labelled estradiol offer the possibility to study the presence of estrogen receptors (ER) in both primary and tumour metastasis, and may be a useful tool in the therapeutic management and prognostic evaluation of breast cancer.

Already back to 1988, Mintun et al. [25] pioneered *in vivo* molecular imaging of breast cancer with PET using the estrogen receptor ligand 16*α*-[^18^F]-fluoro-17*β*-estradiol (^18^F-FES) in a set of 13 patients with primary breast tumours. They found an excellent correlation between uptake of ^18^F-FES and estrogen-receptor concentration measured in vitro after excision of the lesions.

Nowadays, whole-body PET with ^18^F-FES can be seen as a unique method to noninvasively obtain molecular information of ER expression. No other procedure can provide information on a whole body basis of the ER status in metastasic breast cancer. It is well known that ER expression at metastasic sites might not be the same as the ER expression at the primary disease due to relatively common phenotypic changes. In any case, loss of ER expression is more common than gain of ER expression.

Many different studies have shown that ^18^F-FES PET can reliably detect ER-positive tumour lesions and that ^18^F-FES uptake correlates with ER expression as measured by immunohistochemical methods. Furthermore, low ^18^F-FES uptake has been reported to be a strong predictor for failure of antihormonal therapy. For a comprehensive review of ^18^F-FES PET, see [26] and references therein.

Imaging ER expression by PET can be used to identify, characterize, and follow treatment response to multiple lesions in the same patient. ^18^F-FES PET also has great potential for evaluating ER activity in metastasic breast cancer, in which patients have many bone lesions that are difficult to biopsy and prone to false-negative ER by immunohistochemistry [27].

Kurland et al. [28] have recently analysed between-patient and within-patient variability in ER binding by ^18^F-FES-PET and have shown that both ^18^F-FES uptake and the ^18^F-FES/FDG ratio varied greatly between patients but were usually consistent across lesions in the same scan. These results seem to provide a reasonable summary of some kind of “synchronous ER expression” for most patients. However, imaging the entire disease burden remains important to identify the subset of patients with mixed uptake, who may be at a critical point in their disease evolution. These results are also supported by van Kruchten et al. [29] that have shown whole-body imaging of ER expression with ^18^F-FES-PET as a very valuable additional diagnostic tool when standard workup is inconclusive. With the exception of liver metastases, ^18^F-FES PET can be used to support therapy decisions by improving diagnostic understanding and providing information on ER status of tumour lesions.

In a very thorough study with 312 ^18^F-FES-PET scans in 239 patients with documented ER positive primary breast cancer, Peterson et al. [30] demonstrated that ^18^F-FES imaging protocols may be simplified without sacrificing the validity of the results, as calculation of ^18^F-FES SUV should be sufficient to assess tracer uptake for the purpose of inferring ER expression.

## 7. Progesterone Receptors

Hormone-sensitive breast cancer is less aggressive than hormone-resistant disease; hormone-sensitive disease occurs more commonly in postmenopausal women and is characterized by longer disease-free and overall survival.

The presence of the progesterone receptor (PR) increases the likelihood of hormone responsiveness, while progesterone receptor-negative tumours are less responsive to therapy, perhaps suggesting that PR may be necessary for adequate therapeutic outcome. Furthermore, the cross-relationship between estrogen and progesterone receptors—being the former a key transcription factor for the activation of the latter—could suggest that the estrogen response pathway may not be functional in these tumours. In this scenario, noninvasive detection and quantification of PR positive or PR negative lesions would be of enormous value, especially considering that discordance of hormone receptor status between the primary tumour and metastatic disease is not uncommon. This difference may influence patient prognosis and response to therapy. Dehdashti et al. [31] have recently used in humans for the first time a fluorine-18-labelled PR-specific ligand and shown that it can be used to assess the PR status of individual breast cancer lesions. However, no significant correlation was demonstrated between the SUVmax and distribution volume ratio for the tracer uptake and receptor status, likely because of small sample size.

## 8. Radiolabelled Derivatives of P-Glycoprotein Substrates and Inhibitors

Resistance to multiple chemically different drugs is a well-known phenomenon in oncology. Even though the exact cause remains elusive and is multifactorial, membrane proteins that actively remove drugs from the cell are known to play a relevant role.

The best characterized of such drug-resistance proteins is P-glycoprotein (Pgp) (also known as multidrug-resistance protein 1 and ABCB1), a member of the ATP-binding cassette transporters family, which transports substrates across the cell membrane in different conditions. Another relevant member of this family is the breast cancer resistance protein (BCRP, also known as ABCG2). Several PET radiotracers for visualization of Pgp have been described so far [32–36] including carbon-11- and fluorine-18-labelled derivatives of Pgp substrates such as verapamil or loperamide and Pgp inhibitors such as elacridar and tariquidar. The former permit *in vivo* visualization of Pgp function, while the latter can be understood as surrogate markers of Pgp expression levels.

In any case, few Pgp-directed PET tracers have been used in the clinical setting in humans, being probably the paper by Kurdziel et al. [37] describing the human dosimetry and tumour distribution of ^18^F-fluoropaclitaxel in breast cancer patients one of the more recent reports. These authors show that, albeit in a very small series with only three patients, tumour accumulation of the radiotracer could be detected in all cases and that ^18^F-fluoropaclitaxel distribution could be used as a surrogate biomarker for paclitaxel and potentially other chemotherapeutic agents.

## 9. Integrin Ligands

Integrins are obligate heterodimeric proteins involved in cell-cell interaction, interaction of cells with the extracellular matrix, signal transmission, and apoptosis. They are key components of the cell machinery involved in cell signalling, shape, and motility and are part of the complex transduction mechanisms that permit cells to be aware of the changes in their surrounding environment and also participate in the communication of the intracellular changes to the outside.

The *α*_*v*_ *β*_3/5_ subclasses of the integrin family are of particular interest as they are upregulated in tumour neovasculature and on several types of tumour cells (including breast cancer), making them a potentially valuable diagnostic tool. Furthermore, an association between expression of *α*_*v*_ *β*_3/5_ and relapse-free survival in breast cancer has been reported [38], suggesting a prognostic value of imaging such receptors.

Integrin *α*_*v*_ *β*_3_ has been demonstrated to be involved in tumour transformation, angiogenesis, local invasiveness, and metastatic potential. A number of different positron emitter-labelled ligands have been developed to image integrins, and some have already been used in breast cancer patients. Among them, arginine-glycine-aspartic acid (RGD) peptide ligands have high affinity for these integrins and can be radiolabeled for PET imaging of angiogenesis or tumour development [39].

The ability to noninvasively visualize and quantify *α*_*v*_ *β*_3_ integrin expression via RGD will provide new opportunities to document tumour integrin levels, more appropriately select patients who are candidates for antiangiogenic treatment and monitor treatment effectiveness in patients with integrin-positive findings.

**Figure 5:**
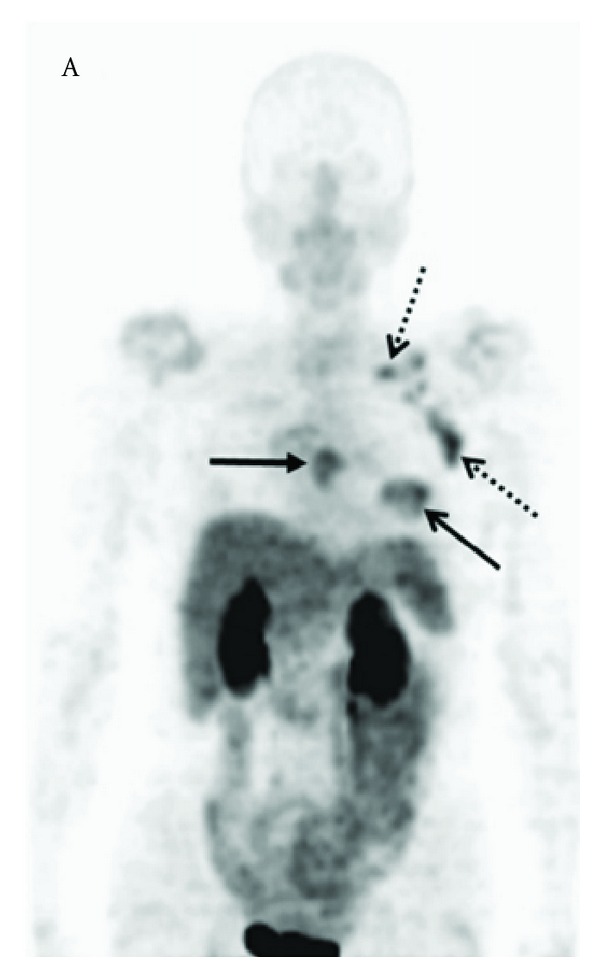
Maximum-intensity projection of ^18^F-galacto-RGD PET in a 70-year-old patient with invasive ductal breast cancer of left breast (arrow, open tip), axillary lymph-node metastases on left side (arrow, open tip, dotted line), an osseous metastasis to the sternum (arrow, closed tip). Reprinted by permission of the Society of Nuclear Medicine from Beer et al. [40], Figure 2.

Back to 2008, Beer et al. [40] reported the use of ^18^F-Galacto-RGD in a group of 16 breast cancer patients and demonstrated tracer uptake in all primary tumour lesions and metastases (Figure 5) although ^18^F-Galacto-RGD uptake in the lesions was very heterogeneous in all cases, suggesting elevated but widely varying levels of *α*_*v*_ *β*_3_ expression in human breast cancer. Immunohistochemical analysis revealed that the detected signal represented a mixture of tracer binding on neovasculature and on tumour cells.

More recently, Kenny et al. [41] have used the cyclic peptide-polymer conjugate ^18^F-fluciclatide (^18^F-AH111585), an aminooxy-functionalized double-bridged RGD derivative with optimized stability. ^18^F-fluciclatide could be used to detect primary and metastatic breast cancer lesions and could be of value for imaging tumours and for pharmacodynamic monitoring of antiangiogenic therapies. In any case, tumour uptake largely varied among individuals and different tumour types, and even between tumours of the same type within one patient. Tomasi et al. [42] have focused on the best quantification approach to analyse the kinetics of ^18^F-fluciclatide in breast cancer patients.

Other RGD-derived radioligands based on the dimeric RGD moiety such as [^18^F]FPPRGD2 have also been recently tested in humans [43]. In addition, a quite large number of different derivatives targeted to image the expression of *α*_*v*_ *β*_3_ integrin have been developed in the last years and tested *in vivo* in small animals, including some very promising gallium-68 labelled RGD peptides [44].

## 10. Monoclonal Antibodies

Immuno-PET has largely been seen as an exciting option for better understanding the *in vivo* behaviour and efficacy of monoclonal antibodies (mAbs) in individual patients. Very many papers have been written to describe the benefits of these visible magic-bullet approach—see [45, 46] and references therein—, and many radiolabelled antibodies and derivatives have been used for small animal imaging.

For labelling intact antibodies with a low-clearance kinetics, longer-lived radioisotopes such as zirconium-89 or iodine-124 are the election of choice, while for radiolabelling mAbs fragments or constructs (minibodies, affibodies, diabodies, etc.) which are more rapidly cleared from the body, shorter-lived positron emitters such as gallium-68 or copper-64 might be ideally suited.

Currently, 12 mAbs have been approved by the FDA for the treatment of cancer, all being intact mAbs [47]. Seven of the mAbs have been approved for the treatment of hematological malignancies, and five for the therapy of solid tumours [48]. However, it has only been very recently that several crucial concurrent achievements have been obtained to allow broad-scale application of (mainly) ^89^Zr-immuno-PET in clinical mAb development and applications. The aforementioned advances are related with the production and commercial availability of ^89^Zr for clinical use and the development of chelates for facile and stable coupling of ^89^Zr to mAbs.

Overexpression of HER2/neu in breast cancer is correlated with a poor prognosis. It may vary between primary tumours and metastatic lesions and change during the treatment. Therefore, there is a need for a new means to assess HER2/neu expression *in vivo*. A ^68^Ga-labeled HER2 derivative affibody has been used to monitor HER2/neu expression in breast cancer xenografts [49] and preliminary results suggest that it could be sensitive enough to detect different levels of HER2/neu expression *in vivo*. The mAb trastuzumab targets the human epidermal growth factor receptor kinase (ERBB or HER) signaling network and has a history as a therapeutic for metastatic or adjuvant treatment of oncological disease. Upregulation of the ERB2 (HER2/neu) receptor has been associated with metastasis and poor prognosis in many cancers, including breast cancer. Specifically, HER receptors stimulate growth and regulate survival and differentiation. Because of variable HER2/neu receptor expression over time, noninvasive and dynamic measurement methods would be ideal for monitoring potential treatment and disease prognosis.

However, there is only one paper published so far describing the use of immuno-PET in humans and just in fourteen patients [50] and an additional case report [51], but it concludes that PET scanning after administration of ^89^Zr-trastuzumab allows visualization and quantification of uptake in HER2-positive lesions in patients with metastatic breast cancer. The PET images obtained with ^89^Zr-trastuzumab revealed high spatial resolution and a good signal-to-noise ratio. Excellent tumour uptake and visualization of HER2-positive metastatic liver, lung, bone, and brain tumour lesions were obtained. ^89^Zr-trastuzumab PET visualized bony metastatic disease. These early studies show great promise for the potential of ^89^Zr-trastuzumab in immuno-PET.

## 11. Exotic Sugar-Like Tracers

A number of different tracers targeting diverse cellular biochemical mechanisms involved in breast cancer have been developed, synthesised, and tested in breast cancer xenograft models. Although an exhaustive list is well beyond the scope of this paper, it is worth citing a couple of very recent articles describing the use of sugar-like derivatives as alternatives to FDG for tumour imaging: a fluorine-18-labelled inositol derivative [52] and a fluorine-18-labelled fructose derivative used to image GLUT5 transporter [53].

## Figures and Tables

**Table 1:** Nuclear characteristics of different selected positron emitters of interest in PET imaging. The maximum positron energy is related to the maximum theoretical resolution using this radionuclide, in such a way that the smaller the positron emission energy, the better the resolution than can be achieved. The branching ratio refers to the approximate number of decay events in which a positron is emitted (this value is 100% for pure positron emitters).

Isotope	Half life	Maximum positron energy (MeV)	Branching ratio (%)
Carbon-11	20 min	0.96	~100
Fluorine-18	110 min	0.63	~100
Copper-64	12.7 h	0.58	~19
Gallium-68	68 min	1.89	~88
Bromine-76	16.2 h	3.94	~100
Zirconium-89	3.26 days	0.89	~23
Iodine-124	4.2 days	2.135	~23
